# Comparison of Polyglactin 910 With Barbed Sutures for Vaginal Cuff Closure Time in Patients Undergoing Total Laparoscopic Hysterectomy

**DOI:** 10.7759/cureus.83708

**Published:** 2025-05-08

**Authors:** Mahendra G, Preethi Maski, Subbappa K, Ramesh Babu

**Affiliations:** 1 Department of Obstetrics and Gynecology, Adichunchanagiri Institute of Medical Sciences, B.G Nagara, IND

**Keywords:** barbed suture, polyglactin 910 suture, post-operative pain, total laparoscopic hysterectomy, vaginal cuff complications, vault closure time

## Abstract

Background

Total hysterectomy is among the most performed gynecological surgeries globally, with a significant shift toward laparoscopic approaches for benign uterine conditions. While laparoscopic hysterectomy offers several benefits, such as reduced pain, shorter recovery, and fewer complications, challenges in laparoscopic suturing of the vaginal cuff remains. The introduction of barbed sutures, which eliminate the need for knotting, has been proposed as an effective alternative to traditional suturing.

Methods

A prospective, two-arm, randomized, single-center study was conducted at Adichunchanagiri Institute of Medical Sciences, Karnataka, India, from May to October 2024. Sixty adult women, aged 30-75 years, scheduled for total laparoscopic hysterectomy (TLH) due to benign uterine pathologies were enrolled. Participants were randomly assigned to one of two groups using a pre-sealed envelope: the unidirectional barbed suture group with a triangular end stopper and a black needle or the polyglactin 910 suture group with a black needle. Primary and secondary outcomes included comparison of vault closure time, total operative time, postoperative pain (measured by the visual analog scale), intraoperative handling of sutures, and vaginal cuff-related complications.

Results

The barbed suture group had a significantly shorter mean vault closure time (6.94 ± 3.39 minutes vs 11.75 ± 4.59 minutes; p = 0.00002) and total operative time (102.27 ± 22.27 minutes vs 131.10 ± 24.99 minutes; p = 0.000015) as compared to the polyglactin 910 suture group. Postoperative pain levels and intraoperative handling properties of the sutures were comparable between the two groups. No vaginal cuff-related complications were observed in either group.

Conclusion

The barbed suture significantly reduced vault closure time and total operative time compared to the polyglactin 910 suture, suggesting that it may enhance the efficiency of laparoscopic vaginal cuff closure. However, both suture types demonstrated comparable postoperative pain and complication rates. These findings support the use of both sutures as safe and effective options for TLH, with the barbed suture offering advantages in vault closure time and overall surgical time.

## Introduction

Total hysterectomy is among the most performed gynecological surgeries worldwide. The percentage of hysterectomy surgeries performed on women between the ages of 40 and 80 has risen from 10% to 38% over time, with a noticeable trend toward laparoscopic techniques for benign cases [1,2]. The prevalence of hysterectomy surgery in India is approximately 17 per 1000 ever-married women [3], with an overall prevalence rate of 3.2% [4]. Common reasons for performing a hysterectomy include tumors, myomas, dysfunctional uterine bleeding, and endometriosis [2].

There are three primary approaches for performing a hysterectomy: abdominal, vaginal, and laparoscopic. The American Congress of Obstetricians and Gynaecologists strongly recommends minimally invasive techniques, which have gained traction because of the advantages they offer [5].

Total laparoscopic hysterectomy (TLH) has become a widely accepted alternative to traditional abdominal hysterectomy for managing benign uterine conditions. Its advantages include reduced pain, shorter hospital stays, quicker healing, less intraoperative blood loss, fewer infections, and better cosmetic outcomes. As a result, laparoscopic surgery has emerged as the standard for treating both benign and malignant uterine conditions, offering a safer option than traditional methods. However, TLH requires specialized techniques and presents challenges. One such challenge is intracorporeal suturing and knotting of the vaginal cuff, which requires precision in a confined space with limited visibility and remains a key hurdle during the procedure [2,6-8]. Various techniques and suture materials have been developed to overcome this challenge.

Currently, the polyglactin 910 suture is widely used for the closure of the vaginal cuff by continuous interlocking suturing and knotting in TLH [8]. In recent years, barbed sutures have been introduced to simplify the suturing process by eliminating the need for knotting, thereby reducing the time and related complications. These are available in various configurations such as unidirectional or bidirectional, absorbable or non-absorbable, and with different anchoring elements (loop or anchor) to fix the suture in the tissue. They also come in various sizes, lengths, and needle combinations [2]. The TRUMAS range of sutures, developed by Healthium Medtech Limited, Bengaluru, India, comes with shorter suture lengths, convenient for intracorporeal suturing and knotting, and with an anti-reflective black needle to enhance visibility during laparoscopic procedures and reduce glare and improve contrast. Currently, there are no studies published on these kinds of sutures and needle types for vaginal cuff closure during TLH. Hence, this study aimed to compare the polydioxanone TRUMAS unidirectional barbed suture with a triangular end stopper with a black needle with the TRUMAS Polyglactin 910 suture with a black needle, focusing on the comparison of vault closure times during TLH.

This article was previously presented as an oral presentation at the 67th All India Congress of Obstetrics and Gynaecology (AICOG), Mumbai, held on January 11, 2025.

## Materials and methods

Study design

This prospective, two-arm, randomized, single-centre study was conducted at the Department of Obstetrics and Gynecology of Adichunchanagiri Institute of Medical Sciences, B.G Nagara, Karnataka, India, from May 2024 to October 2024. The primary objective was to compare the vault closure time during TLH between a unidirectional barbed suture with a triangular end stopper with a black needle and a polyglactin 910 suture with a black needle. Secondary objectives included evaluating total operative time from first incision till closure, postoperative pain, intraoperative handling properties of the suture materials and vaginal cuff-related complications. Participants were randomised to one of the two groups using pre-sealed envelopes.

Ethical considerations

The study was registered with the Clinical Trial Registry of India (CTRI/2024/05/066985; date: 08/05/2024) and approved by the Ethics Committee of Adichunchanagiri Institute of Medical Sciences (approval number: ECNEW/INST/2023/KA/0382, dated 06/04/2024). The study adhered to the ethical principles outlined in the Declaration of Helsinki, ICH Good Clinical Practice (GCP) E6 R2 guidelines, ISO 14155:2020, Indian Medical Devices Rules (MDR) 2017, New Drugs and CT Rules 2019, and relevant Indian regulations.

Sample size calculation

The required sample size was estimated based on the standardized mean difference (SMD) in vaginal cuff suturing time reported in a meta-analysis by Bogliolo et al. [9], which demonstrated a significant reduction in suturing time with barbed sutures compared to conventional sutures (SMD = -0.96; 95% CI: -1.26 to -0.70; p < 0.001).

Using Cohen’s d: 

$n = \frac{\{2 (Z_{1-\alpha/2} + Z_{1-\beta})^2 \}}{d^2}$

Where:

$d = \sigma \Delta$: standardized effect size (SMD from the meta-analysis = 0.96)

Using:

$Z_{1-\alpha/2} = 1.96$

$Z_{1-\beta} = 0.84$

$d = 0.96$

Using this effect size (Cohen’s d = 0.96) and assuming a two-sided alpha level of 0.05 with 80% power, the minimum sample size required was calculated to be 18 participants per group. To improve the statistical power and account for potential variability, we included 30 participants in each group in the current study.

Study setting and participants

The study included 60 adult women aged 30 to 75 years, diagnosed with benign uterine pathologies, such as abnormal uterine bleeding and pelvic inflammatory disease, and scheduled for TLH.

Inclusion and exclusion criteria

The study included women aged 30 to 75 years who were diagnosed with benign uterine pathologies, such as abnormal uterine bleeding and pelvic inflammatory disease, who were scheduled for TLH, and who agreed to provide written informed consent.

Exclusion criteria included women undergoing total abdominal or vaginal hysterectomy, patients with contraindications to laparoscopic surgery, patients with malignancy, coagulation disorders, or those requiring a prolapse repair surgery.

Study procedure

All procedures were performed by two experienced laparoscopic gynecologic surgeons. Both surgeons were equally involved in operating on participants from both study arms to minimize surgeon-related variability. For every participant, demographic information was gathered, including age, parity, body mass index (BMI), occupation, and educational attainment. Blood tests, pap smears, endometrial aspirations, imaging, physical examinations, history-taking, and other standard procedures were all part of the preoperative evaluations. Prophylactic preoperative antibiotics were given, and all procedures were conducted under general anaesthesia. A 10-mm trocar was placed directly above the umbilicus, and two 5-mm trocars were inserted in the left and right lower quadrants of the abdomen, and one 5-mm trocar was inserted at the midclavicular plane 5 cm above the previous port on the left side. A myoma screw was used to move the uterus, and bipolar coagulation was used to secure the vascular pedicles. The bladder was moved down to about 1.5 cm below the anterior colpotomy incision after being bluntly detached from the cervix and upper vagina. The monopolar needle was used to separate the cervix from the vagina. Vaginal cuff suturing was then done laparoscopically. If there was persistent oozing along the anterior vaginal margin, distal to the cuff and adjacent to the bladder mobilization site, hemostat oxidized regenerated cellulose was used (Clinicel Knitted, Healthium Medtech).

In the non-barbed suture group, the vaginal cuff was closed using the TRUMAS polyglactin 910 suture with a black needle, starting from the right side and ending on the left. The suture was tied using an extracorporeal knot, approximating the anterior and posterior vaginal epithelia along with their underlying fascia. In the barbed suture group, the vaginal cuff was approximated using the TRUMAS unidirectional absorbable barbed suture with a triangular end stopper and black needle, beginning on the right side and ending on the left. The triangular end stopper facilitates continuous suturing after the first bite, eliminating the need to hook the loop with the needle to secure the knot. The barbs on the suture hold the anterior and posterior vaginal epithelia and their underlying fascia in place, maintaining approximation without the need for knotting.

Outcome measures

Primary outcome: To compare the vault closure time during TLH in both groups (measured on the day of surgery).

Secondary outcomes: 1) To evaluate the total operative time from first incision till closure in both groups (measured on the day of surgery); 2) to evaluate the postoperative pain measured using a 10-point visual analog scale (VAS) score (measured on the day of surgery, postoperative day 2, day of discharge, and postoperative days 7 and 21). The VAS is a numeric scale ranging from 0 (low) to 10 (high) used for pain assessment; 3) to evaluate intraoperative handling properties of both suture materials assessed using the Product Usage Assessment Scale, which is a 5-point scale, with 5 being excellent and 1 being poor (measured on the day of surgery); To assess vaginal cuff-related complications, such as dehiscence, hematoma, or abscess (measured on the day of surgery, postoperative day 2, day of discharge, and postoperative days 7 and 21).

Statistical analysis

The demographic data, including age, education level, parity, and co-morbidities, were summarized using descriptive statistics, and group comparisons were performed using the independent t-test for continuous variables and the chi-square test for categorical variables. For the comparison of vault closure time and total operative time between the two groups, the independent t-test was used. Postoperative pain levels and intraoperative handling characteristics of sutures were also analyzed using independent t-tests to assess the differences between the two groups. All statistical analyses were performed using Microsoft Excel (Microsoft Corporation, Redmond, WA, US), and a P-value of less than 0.05 was considered statistically significant for all tests.

## Results

**Figure 1:**
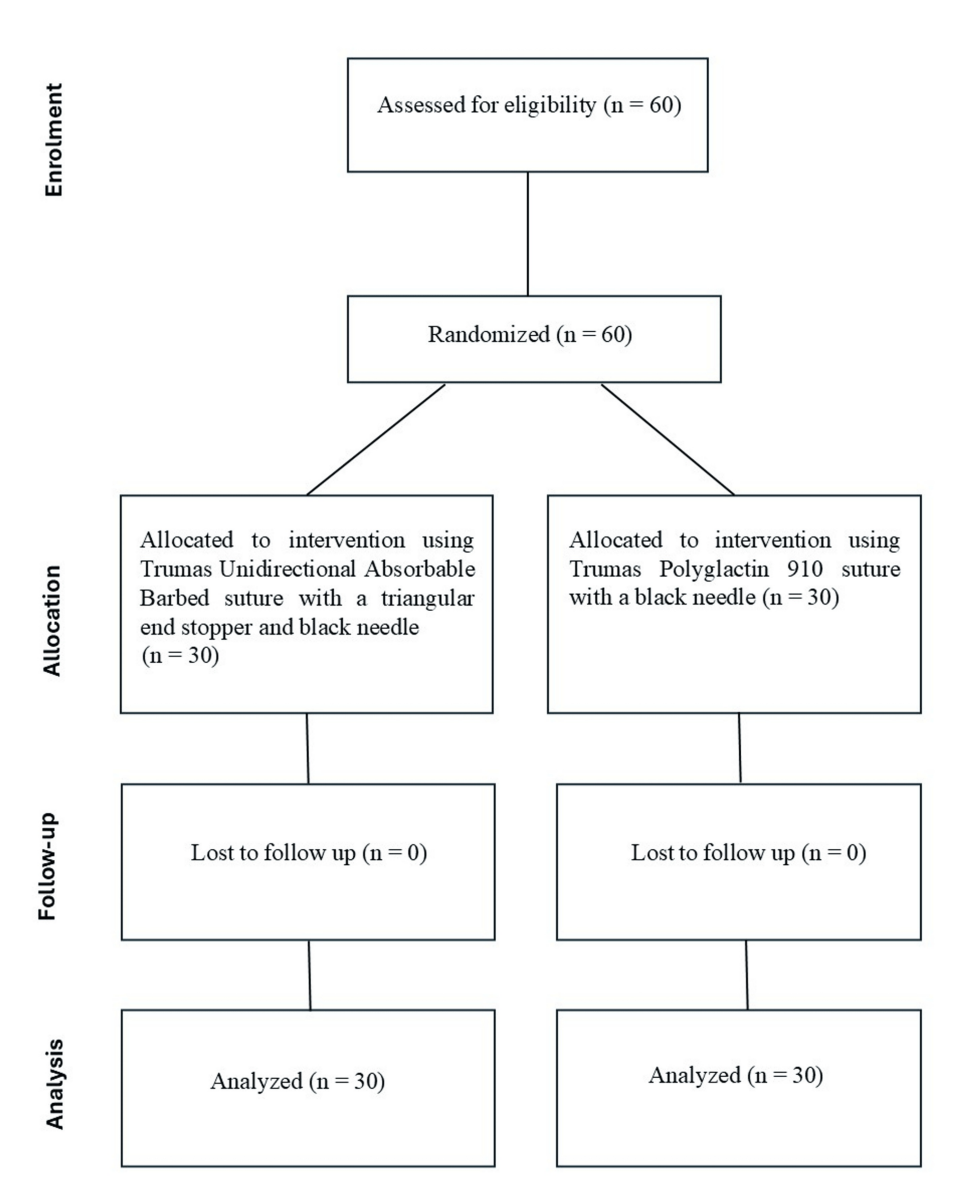
Participant flow

A total of 60 patients were enrolled in the study, with 30 patients assigned to each of the two groups (Figure 1).

No significant differences were observed between the two groups in terms of age, parity, and co-morbidities. The mean age of the Barbed Suture group was 44.87 ± 4.52 years, while the Polyglactin 910 Suture group had a mean age of 43.63 ± 3.70 years. The majority of participants in both groups were multigravida, with 83.33% in the Barbed Suture group and 90% in the Polyglactin 910 Suture group. Regarding co-morbidities, both groups had similar rates of hypertension, diabetes mellitus, hypothyroidism, and hyperthyroidism (Table 1).

**Table 1:** Demographic variables Continuous data are presented as Mean ± SD, and categorical data as n (%). ^#^P-values are based on the independent samples t-test One patient in the Barbed Suture group had both hypertension and hypothyroidism. This patient is captured under both headings.

Variable	Barbed Suture group (n=30)	Polyglactin 910 Suture group (n=30)	P-value^#^
Mean Age (years)	44.87 ± 4.52	43.63 ± 3.70	0.25
Education Level, n (%)			
High School	19 (63.33%)	15 (50%)	0.042 (Statistically significant)
Pre-High School	5 (16.66%)	1 (3.33%)	
Pre-University	6 (20%)	14 (46.66%)	
Parity, n (%)			
Primigravida	4 (13.33%)	3 (30%)	0.543
Multigravida	25 (83.33%)	27 (90%)	
Nulligravida	1 (3.33%)	0	
Co-morbidities, n (%)			
Nil	24 (80%)	22 (73.33%)	0.568
Hypertension	3 (10%)	3 (10%)	
Diabetes Mellitus	3 (10%)	1 (3.33%)	
Hypothyroidism	1 (3.33%)	3 (10%)	
Hyperthyroidism	0	1 (3.33%)	

**Figure 2:**
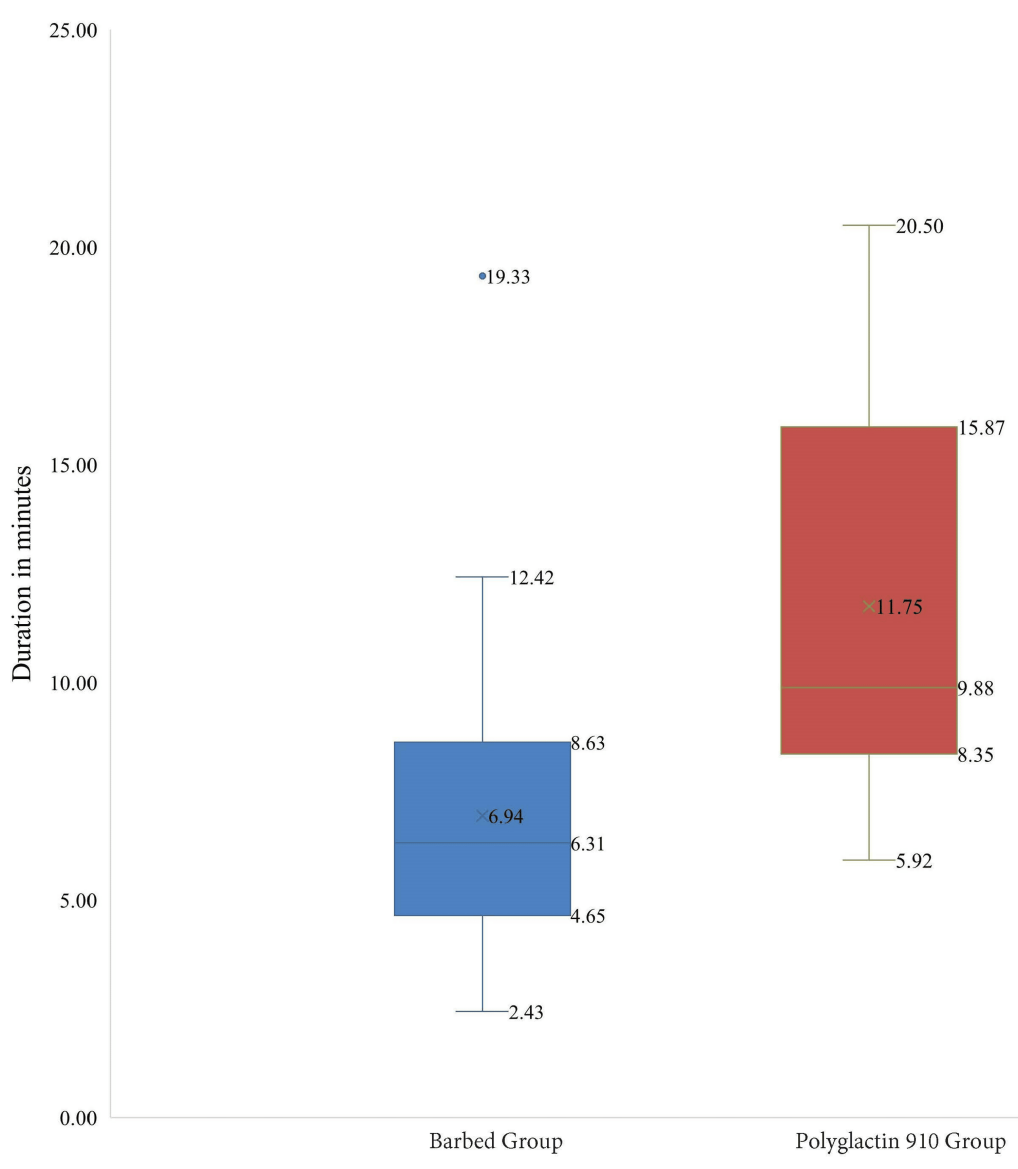
Comparison of vault closure times The horizontal lines represent the maximum and minimum values, the cross (×) symbols represent the mean, and the dot (•) symbols represent the outliers.

The mean vault closure time was significantly shorter for the Barbed Suture group (6.94 ± 3.39 minutes) compared to the Polyglactin 910 Suture group (11.75 ± 4.59 minutes), with the independent samples t-test p-value = 0.00002 as shown in Figure 2.

**Figure 3:**
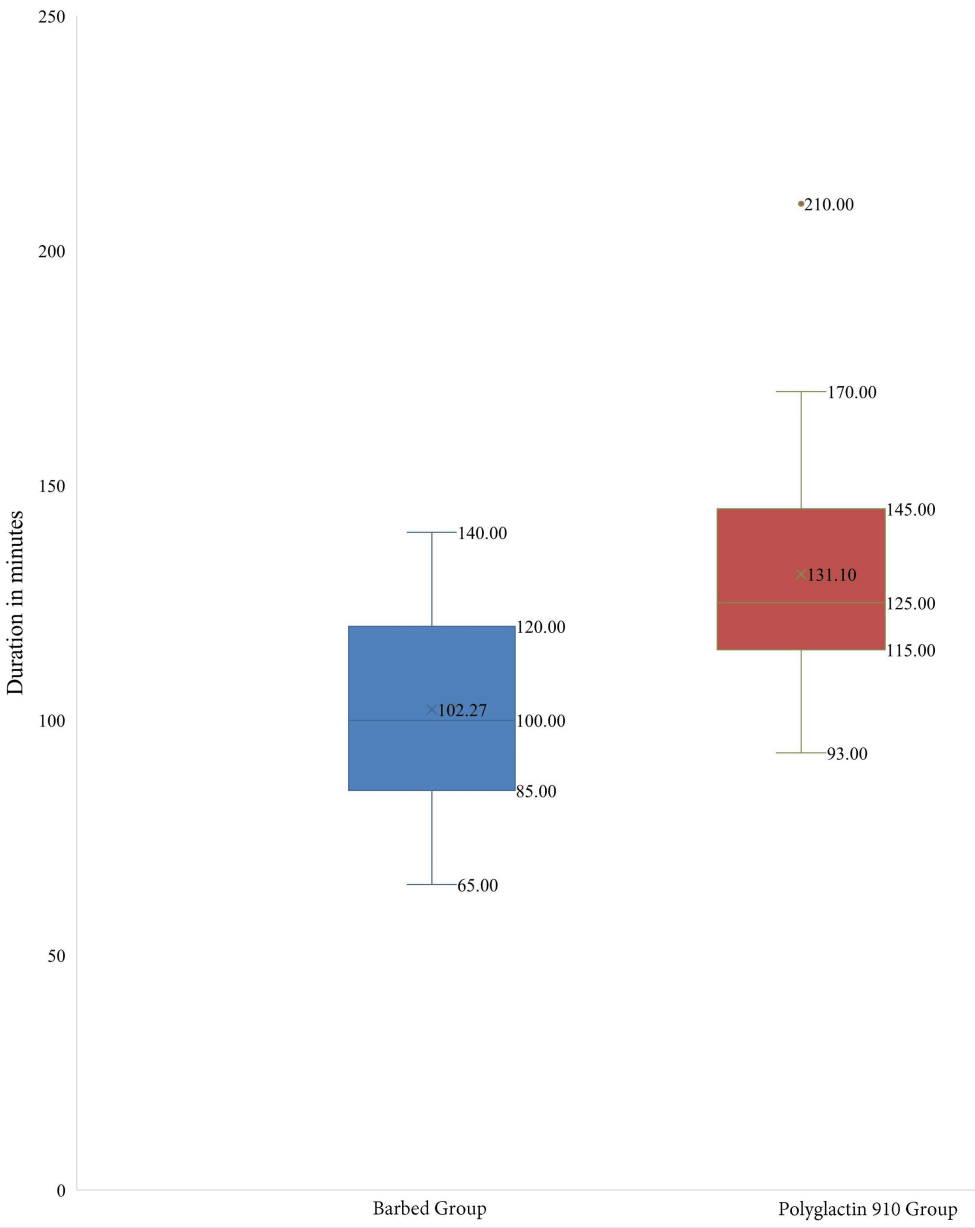
Comparison of total operative time The horizontal lines represent the maximum and minimum values, the cross (×) symbols represent the mean, and the dot (•) symbols represent the outliers.

The Barbed Suture group also had a mean operative time of 102.27± 22.27 minutes, while the Polyglactin 910 Suture group had a mean time of 131.10± 24.99 minutes (independent sample t-test p-value = 0.000015). This indicates a statistically significant difference in favor of the Barbed Suture group (Figure 3).

The postoperative pain was assessed using the VAS score at various time points, which revealed no significant differences, indicating that both suture types resulted in comparable levels of postoperative pain at all measured time points. However, it is notable that both groups' pain scores decreased steadily at all subsequent time points (Table 2).

**Table 2:** Comparison of postoperative pain at various time points Continuous data are presented as Mean ± SD. ^#^P-values are based on the independent samples t-test.

Time points	Mean scores of the Barbed Suture group (n=30)	Mean scores of the Polyglactin 910 Suture group (n=30)	P-value^#^
Day of Surgery	6.97 ± 0.81	6.57 ± 1.17	0.127
Postop Day 2	5.77 ± 0.63	5.63 ± 0.81	0.478
Day of Discharge	3.83 ± 0.75	3.63 ± 0.49	0.224
Postop Day 7	3.27 ± 0.69	3.63 ± 1.22	0.156
Postop Day 21	1.60 ± 0.50	1.77 ± 1.25	0.500

Intraoperative handling properties of sutures between the groups using the Product Assessment scale (5-point scale, with 5 being excellent and 1 being poor), revealed no significant differences across all assessed parameters (Table 3).

**Table 3:** Comparison of intraoperative handling properties of sutures using the Product Usage Assessment scale Continuous data are presented as Mean ± SD. ^#^P-values are based on the independent samples t-test.

Parameter	Mean scores of the Barbed Suture group (n=30)	Mean scores of the Polyglactin 910 Suture group (n=30)	P-value^#^
Reduction in Eye Fatigue due to Black Needle	4.00 ± 0.74	3.97 ± 0.61	0.850
Depth of Perception with Needle	3.63 ± 0.56	3.63 ± 0.67	1
Maneuverability of Needle	4.27 ± 0.64	4.20 ± 0.66	0.693
Ease of Introduction of Needle into Trocar	4.40 ± 0.50	4.17 ± 0.59	0.104
Grip Provided by Needle	4.37 ± 0.72	4.03 ± 0.72	0.077
Ease of Suture Usage	3.83 ± 0.38	3.83 ± 0.59	1
Ease of Retrieval from Package	4.53 ± 0.57	4.43 ± 0.57	0.499
Overall Satisfaction with Suture	4.17 ± 0.53	4.23 ± 0.50	0.619

Persistent oozing along the anterior vaginal margin was noted in one patient from the Barbed Suture group and three patients of the Polyglactin 910 Suture group, and hemostat oxidized regenerated cellulose was used (Clinicel Knitted) in all these four cases. Additionally, no vaginal vault-related complications were noted in either group, indicating that both suture types were associated with a complication-free outcome.

## Discussion

Barbed sutures have been demonstrated to be as safe and well-tolerated as traditional sutures, with the added advantage of significantly reducing operative time during laparoscopic vaginal cuff closure. In addition, they contribute to decreased intraoperative blood loss, reduced risk of vaginal cuff dehiscence, and lower overall surgical difficulty, without increasing hospital stay or postoperative complications. These sutures feature self-retaining barbs that anchor securely within the tissue, facilitating effective tissue approximation and preventing slippage, thereby eliminating the need for knot tying.

Although barbed sutures are relatively more expensive, they are widely regarded as safe and easy to use. Their hemostatic property and ease of application make them ideal for laparoscopic surgeries, including hysterectomy. These sutures maintain consistent tensile strength across the entire wound length, allowing for continuous suturing and easier hemostasis. Furthermore, barbed sutures are considered quicker and easier to learn, making them a favourable option for both experienced surgeons and trainees, resulting in faster closure times and potential cost savings in the operating room [1,2,7,8].

In the present study, a significant reduction in vaginal vault closure time was observed with the use of continuous barbed sutures (6.94 ± 3.39 minutes) compared to conventional Polyglactin 910 sutures (11.75 ± 4.59 minutes; p = 0.00002). The reduced closure time is likely attributable to the unique design of the TRUMAS unidirectional barbed suture, specifically the triangular end stopper, which negates the need for securing the first knot, and the self-locking barbs that maintain approximation without further knotting. This finding is consistent with previous studies demonstrating that barbed sutures reduce vault closure time during laparoscopic hysterectomy [1,7-14]. 

Multiple meta-analyses have reinforced the safety and effectiveness of barbed sutures, underscoring their advantage in reducing suturing time compared to conventional sutures during vaginal cuff closure [9,15-18]. In our study, the Barbed Suture group exhibited a significantly lower mean operative time (102.27 ± 22.27 minutes) compared to the Polyglactin 910 Suture group (131.10 ± 24.99 minutes; p = 0.000015). These findings are in line with results reported by Zhou Y et al., Nawfal et al., Kim SM et al., and Karacan T et al., who similarly found shorter operative durations when using unidirectional barbed sutures [19-22].

Concerning postoperative pain, our results showed no significant differences between the two groups at various postoperative time points, indicating comparable levels of patient discomfort regardless of the suture type. This is consistent with findings by Lopez et al. and Khoiwal et al., who also reported similar pain outcomes in both groups [8,23].

Importantly, there were no reported cases of vaginal vault dehiscence, hematoma, abscess, or dyspareunia in either group. This aligns with the findings of Kim JH et al. and Talwar et al., who similarly reported no cases of cuff dehiscence in their studies [7,13]. Moreover, no adverse medical device events or other complications were observed in either group. These outcomes suggest that both barbed and conventional sutures are safe and effective for vaginal cuff closure, offering comparable postoperative safety profiles.

Limitations

Despite the valuable findings of this study, several limitations should be acknowledged. First, the study was conducted at a single center, which may limit the generalizability of the results to other clinical settings with different surgical teams or patient populations. Second, the sample size, though adequate for detecting differences in vault closure and operative times, may not be sufficiently powered to detect rare complications or long-term outcomes such as vaginal cuff dehiscence, chronic pain, or dyspareunia. Third, the study focused exclusively on short-term outcomes, with follow-up limited to 21 days postoperatively. Longer-term follow-up is necessary to evaluate potential delayed complications or differences in healing and tissue integration between suture types. While intraoperative handling properties were assessed using a structured scale, subjective bias cannot be entirely ruled out.

## Conclusions

In conclusion, the TRUMAS unidirectional barbed suture with a triangular end stopper with a black needle suture significantly reduced vault closure time and total operative time compared to the TRUMAS Polyglactin 910 suture with a black needle, supporting its utility in improving the efficiency of laparoscopic vaginal cuff closure. However, both sutures yielded comparable postoperative outcomes, highlighting that both suture types are safe and effective for the TLH procedure. Further research with larger sample sizes may provide additional insights into the long-term outcomes and complications associated with these suture types.
